# Hepatic iron overload, a possible consequence of treatment with imatinib mesylate: a case report

**DOI:** 10.1186/1757-1626-2-7526

**Published:** 2009-05-26

**Authors:** Baidehi Maiti, Sebouh Setrakian, Hamed A Daw

**Affiliations:** 1 Department of Internal Medicine, 18101 Lorain Avenue, Fairview HospitalCleveland, Ohio 44111USA; 2 Department of Pathology, 18101 Lorain Avenue, Fairview HospitalCleveland, Ohio 44111USA; 3 The Cleveland Clinic Cancer Center at Fairview Hospital, 18200 Lorain Avenue, Cleveland, Ohio 44111USA

## Abstract

Imatinib, a tyrosine kinase inhibitor has revolutionized the therapy of Philadelphia chromosome positive chronic myeloid leukemia. Side effects of imatinib include grade 1-4 hepatotoxicity in a subset of patients. We report the case of a 46-year-old male with chronic myeloid leukemia, who developed hepatic hemosiderosis during treatment with imatinib. After ruling out the established congenital and acquired causes of hepatic hemosiderosis, we attribute this to a possible side effect of imatinib therapy. This condition was successfully treated with periodic phlebotomy thus precluding discontinuation of imatinib. To our knowledge, this is the first report of hepatic hemosiderosis most likely consequent to imatinib therapy.

## Introduction

Chronic myeloid leukemia (CML) is a myeloproliferative disorder characterized by the presence of Philadelphia chromosome, which is the translocated 9:22 chromosome giving rise to constitutively active BCR-Abl fusion protein [1,2].

Imatinib mesylate, an orally administered tyrosine-kinase inhibitor, targets this fusion protein and produces anti-proliferative and pro-apoptotic effects on the neoplastic cells containing the translocation. Reported hepatic side effects observed with imatinib range from mild elevation of transaminases, to acute fulminant hepatitis and fatal liver failure [3-4,7]. We report the case of a 46-year-old male with CML who developed high levels of transaminases secondary to hemosiderosis likely induced by imatinib therapy. Intereference by imatinib in the iron metabolism is also suggested by a recent report of intradermal hemosiderosis consequent to imatinib therapy [6].

## Case presentation

This is the case of a 46-year-old Caucasian male who came to us with complaints of mild fatigue and unexplained weight loss of about 28 pounds over 8 month period. Physical exam was remarkable for splenomegaly without any lymphadenopathy. Labs revealed leucocytosis with a white cell count of 63.3 × 103/μl. A manual differential count of peripheral blood smear showed neutrophil 44%, lymphocyte 1%, monocyte 3%, eosinophil 1%, basophil 6%, bands 22%, metamyelocyte 14%, myelocyte 9%. Following diagnosis of chronic phase CML by bone marrow biopsy the patient was started on 400 mg imatinib daily. Within a month of treatment, his white counts dropped from about 63,000/μl to 12,000/μl. Cytogenetic tests showed typical 9:22 translocation in all 20 banded cells examined. 4 of these cells also had an additional Philadelphia chromosome indicating a possible clonal evolution suggestive of accelerated or blast phase crisis of the disease. Thus the dosage of imatinib was increased to 600 mg daily. In another couple of weeks, his white cell count dropped to 3400/μl alongwith a drop of hemoglobin from 13.4 gm/dL to 11.1 gm/dL and platelets from 239,000/ μl to 57,000/ μl. Further drop of WBC count to 1800/ μl over the next 10 days warranted a reduction of imatinib dosage from 600 mg to 400 mg daily. His white count however continued to drop to 1500/ μl when he became febrile and was advised to discontinue imatinib for a couple weeks. His fever resolved with antibiotics and following 2 weeks of discontinuation of imatinib, his white count was back up at 5100/ μl, with absolute neutrophil count of 2700/ μl, hemoglobin 13.9 and platelets 190,000/ μl. The patient was restarted on 400 mg of imatinib daily. He tolerated this dose well for 2 weeks without any drop in cell counts and the imatinib dosage was increased to 500 mg daily. This dose was continued thereafter.

**Figure 1.**
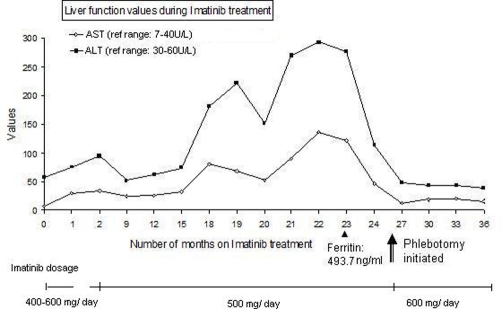
Imatinib causes elevated liver transaminases. Temporal relationship of imatinib therapy with markedly elevated ALT and AST levels secondary to hepatic hemosiderosis. The concurrently elevated serum ferritin level and the time of initiation of treatment with phlebotomy are indicated. Patient was on imatinib 400-600 mg daily during the whole period of time except a brief discontinuation of 2 weeks in the second month due to neutropenic fever. The dosage of imatinib is indicated. The break in the line indicates the 2-week period during which imatinib was discontinued for neutropenic fever.

**Figure 2.**
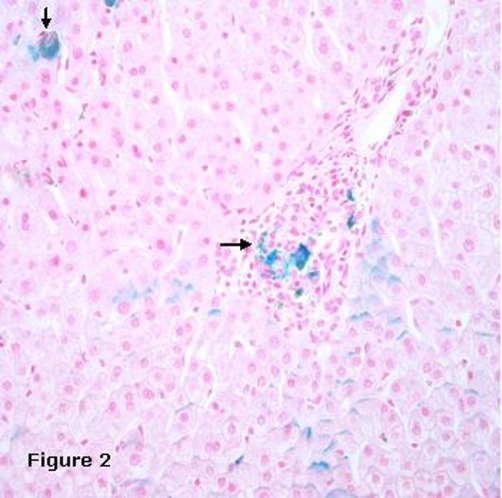
Hepatic hemosiderosis is a likely consequence of imatinib treatment. Liver biopsy with iron stain revealing prominent iron deposition within hepatic parenchymal cells and portal areas (indicated).

By the first 9 months following initial diagnosis the patient was able to achieve and maintain a complete molecular remission as evident by quantitative RT-PCR (reverse transcriptase polymerase chain reaction) of BCR-Abl transcript. His splenomegaly had resolved clinically. It was concerning however that his liver function tests became increasingly abnormal. Although his baseline liver function tests were within normal limits prior to treatment initiation, his ALT (alanine aminotransferase) was noted to be mildly elevated one month after the start of imatinib therapy, became normal after nine months and then again progressively increased to markedly high levels at eighteen months (Figure 1). On the contrary, the AST (aspartate aminotransferase) was first elevated at eighteen months of imatinib therapy and then progressively increased albeit to lesser extents than ALT (Figure 1). At 22 months following imatinib therapy, the AST was at about 3 times the upper limit of reference range while ALT level peaked at approximately 5 times the upper limit of reference range. The strikingly elevated liver enzymes with a normal alkaline phosphatase and bilirubin were suggestive of hepatocellular injury in absence of a cholestatic etiology. With an effort to address the problem without having to discontinue imatinib therapy, we decided to investigate in to the etiology of increased transaminases. Patient's occasional alcohol intake history, normal viral hepatitis serologic studies, alpha fetoprotein level, ceruloplasmin level, magnetic resonance imaging of the abdomen were helpful in ruling out alcoholic liver disease, viral hepatitis, focal nodular hyperplasia, malignant hepatic involvement, Budd-Chiari syndrome and Wilsons disease. Finally, on patient's consent, a liver biopsy with iron stain revealed prominent iron deposition within hepatic parenchymal cells and portal areas as well as mild portal fibrosis (Figure 2). Consistently, the ferritin levels were found to be markedly elevated, while transferrin saturation levels were within normal limits (Figure 1). These findings prompted the genetic analysis for hereditary hemochromatosis (C282Y and H63D loci), which turned out to be negative. Following discussion with patient, phlebotomy was initiated which dramatically brought the liver enzymes back to normal levels (Figure 1). Following the first phlebotomy, the imatinib dosage was increased to 600 mg daily and continued thereafter. Patient had the initial two phlebotomies 3 months apart followed by every 6 months. Patient's liver functions remained within normal limits until his recent follow up at 42 months after initiating imatinib therapy and roughly 15 months following initiation of phlebotomy. Hence the patient was able to maintain imatinib treatment with continued molecular remission, taking recourse only to periodic phlebotomy at 3-6 month intervals. Patient tolerated phlebotomy well with no side effects or anemia.

## Discussion

The discovery of the BCR-Abl translocation in CML culminated in the realization of the dream of targeted therapy in cancer by the small molecule tyrosine kinase inhibitor imatinib [9]. The recently reported result of 6-year Phase III clinical trial confirms the durability of response to imatinib in addition to the declining incidence of adverse events over time [1]. Currently, indications of imatinib include the treatment of CML and gastrointestinal stromal tumor (GIST). Most patients with newly diagnosed chronic phase CML experience complete cytogenetic responses with imatinib treatment, however continuation of treatment is required to prevent molecular or cytogenetic relapse [8]. According to the literature, recurrent liver toxicity was the second most common reason for permanent discontinuation of imatinib therapy [4,5]. Of all the patients undergoing treatment with imatinib, only a minor subset reportedly exhibits hepatotoxicity [10-14]. Grade 1 elevation in transaminases was observed in up to 6-12% of patients and grade3/4 in 3-8% of patients receiving imatinib therapy [3-5]. The cases of suspected imatinib induced hepatotoxicity responded to discontinuation of treatment either promptly or in a delayed manner [4]. In some cases including a case of imatinib induced immune hepatitis, corticosteroids were helpful in resolving the hepatotoxicity and enabling resumption or continuation of imatinib treatment [4,10-15].

In the absence of transfusional iron overload, hepatic hemosiderosis in the aforesaid case with normal transferrin saturation is a possible consequence of imatinib therapy. The elevated liver enzymes were not associated with any discernible infectious or inflammatory condition hence making hemophagocytosis a less likely possibility. While anti-nuclear antibody (ANA) was elevated at 2.3 OD ratio (ref range <1.5 OD ratio), anti-mitochondrial antibody, anti-smooth muscle antibody and alfa-1-antitrypsin antibody were negative. It seems unlikely that the mechanism of iron overload is a result of increased iron absorption or simply a higher intake in way of iron being an ingredient in imatinib mesylate tablets since there was no evidence of iron overload in any other organs including heart, spleen or pancreas. Whether it was secondary to interference of imatinib with heme metabolism in the reticuloendothelial cells remains to be elucidated. In this case report, we made an effort to understand the pathology behind imatinib induced hepatotoxicity and were able to treat the condition successfully by periodic phlebotomy. This is relatively safer than using measures as corticosteroid therapy or stopping imatinib treatment as have been shown to be effective in reversing other forms of imatinib induced hepatotoxicity. Imatinib induced disturbance in the iron homeostasis is also suggested by a recent report of intradermal hemosiderosis as a possible consequence to imatinib therapy, lending further support to our conclusion that the observed hepatic hemosiderosis is most likely a consequence imatinib therapy [6].

## List of abbreviations

ALT: Alanine transaminase
ANA: Anti-nuclear antibody
AST: Aspartate aminotransferase
BCR-ABL: breakpoint cluster region - Abelson
CML: Chronic myeloid leukemia
GIST: Gastrointestinal stromal tumor
RT-PCR: Reverse transcriptase polymerase chain reaction

## References

[bib-001] Soverini S, Martinelli G, Iacobucci I, Baccarani M (2008). Imatinib mesylate for the treatment of chronic myeloid leukemia. Expert Rev Anticancer Ther.

[bib-002] Schwetz B (2001). New treatment for chronic myeloid leukemia. JAMA.

[bib-003] Rodriguez-Frias EA, Lee WM (2007). Cancer chemotherapy I: hepatocellular injury. Clin Liver dis.

[bib-004] Al Sobhi E, Zahrani Z, Zevallos E, Zuraiki A (2007). Imatinib-induced immune hepatitis: case report and literature review. Hematology.

[bib-005] Deininger Michael WN, O'Brien Stephen G, Ford John M, Druker Brian J (2003). Practical management of patients with chronic myeloid leukemia receiving imatinib. J Clin Oncol.

[bib-006] Belasco KT, Miller RA (2007). Intradermal tissue hemosiderosis presenting as slate-gray hyperpigmentation during treatment with imatinib mesylate. J Am Acad Dermatol.

[bib-007] Kong JH (2007). Early Imatinib-mesylate-induced hepatotoxicity in chronic myelogenous leukemia. Acta Haematol.

[bib-008] Hochhaus A (2008). First-Line management of CML: a state of the art review. J Natl Compr Canc Netw.

[bib-009] Goldman JM, Melo JV (2008). BCR-ABL in chronic myelogenous leukemia - how does it work?. Acta Haematol.

[bib-010] Walid S, Ayoub WS, Geller SA, Tran T, Martin P, Vierling JM, Poordad FF (2005). Imatinib (Gleevec)-induced hepatotoxicity. J ClinGastroentero.

[bib-011] James C, Trouette H, Marit G, Cony-Makhoul P, Mahon FX (2003). Histological features of acute hepatitis after imatinib mesylatetreatment. Leukemia.

[bib-012] Kikuchi S, Muroi K, Takahashi S, Kawano-Yamamoto C, Takatoku M, Miyazato A, Nagai T, Mori M, Komatsu N, Ozawa K (2004). Severe hepatitis and complete molecular response caused by imatinib mesylate: Possible association of its serum concentration with clinical outcomes. Leuk Lymphoma.

[bib-013] Ikeda K, Shiga Y, Takahashi A, Kai T, Kimura H, Takeyama K, Noji H, Ogawa K, Nakamura A, Ohira H, Sato Y, Maruyama Y (2006). Fatal hepatitis B virus reactivation in a chronic myeloid leukemia patient during imatinib mesylate treatment. Leuk Lymphoma.

[bib-014] Ohyashiki K, Kuriyama Y, Nakajima A, Tauchi T, Ito Y, Miyazawa H, Kimura Y, Serizawa H, Ebihara Y (2002). Imatinib mesylate-induced hepato-toxicity in chronic myeloid leukemia demonstrated focal necrosis resembling acute viral hepatitis. Leukemia.

[bib-015] Ferrero D, Pogliani EM, Rege-Cambrin G, Fava C, Mattioli G, Dellacasa C, Campa E, Perfetti P, Fumagalli M, Boccadoro M (2006). Corticosteroids can reverse severe imatinib-induced hepatotoxicity. Haematologica.

